# Targeted therapy approaches for epithelial-mesenchymal transition in triple negative breast cancer

**DOI:** 10.3389/fonc.2024.1431418

**Published:** 2024-10-10

**Authors:** Mazharul Haque, Ritis K. Shyanti, Manoj K. Mishra

**Affiliations:** Cancer Research Center, Department of Biological Sciences, Alabama State University, Montgomery, AL, United States

**Keywords:** breast cancer, triple-negative breast cancer, tumor microenvironment, epithelial-mesenchymal transition, regulatory pathway, targeted therapy

## Abstract

Triple-negative breast cancer (TNBC) is distinguished by negative expression of estrogen receptor (ER), progesterone receptor (PR), and human epidermal growth factor receptor 2 (HER2), making it an aggressive subtype of breast cancer and contributes to 15-20% of the total incidence. TNBC is a diverse disease with various genetic variations and molecular subtypes. The tumor microenvironment involves multiple cells, including immune cells, fibroblast cells, extracellular matrix (ECM), and blood vessels that constantly interact with tumor cells and influence each other. The ECM undergoes significant structural changes, leading to induced cell proliferation, migration, adhesion, invasion, and epithelial-to-mesenchymal transition (EMT). The involvement of EMT in the occurrence and development of tumors through invasion and metastasis in TNBC has been a matter of concern. Therefore, EMT markers could be prognostic predictors and potential therapeutic targets in TNBC. Chemotherapy has been one of the primary options for treating patients with TNBC, but its efficacy against TNBC is still limited. Targeted therapy is a critical emerging option with enhanced efficacy and less adverse effects on patients. Various targeted therapy approaches have been developed based on the specific molecules and the signaling pathways involved in TNBC. These include inhibitors of signaling pathways such as TGF-β, Wnt/β-catenin, Notch, TNF-α/NF-κB and EGFR, as well as immune checkpoint inhibitors, such as pembrolizumab, 2laparib, and talazoparib have been widely explored. This article reviews recent developments in EMT in TNBC invasion and metastasis and potential targeted therapy strategies.

## Graphical Abstract

The EMT has profound impact on the TNBC pathogenisity. The factors involved in the metastasis of TNBC due to EMT have been of great concern. The role of these factors in prognosis has enabled researchers to explore it as targeted drug therapies. The aim of this review is to discuss inclusive insights of EMT role in metastasis and the important marker genes and transcription factors to target TNBC and address these challenges.

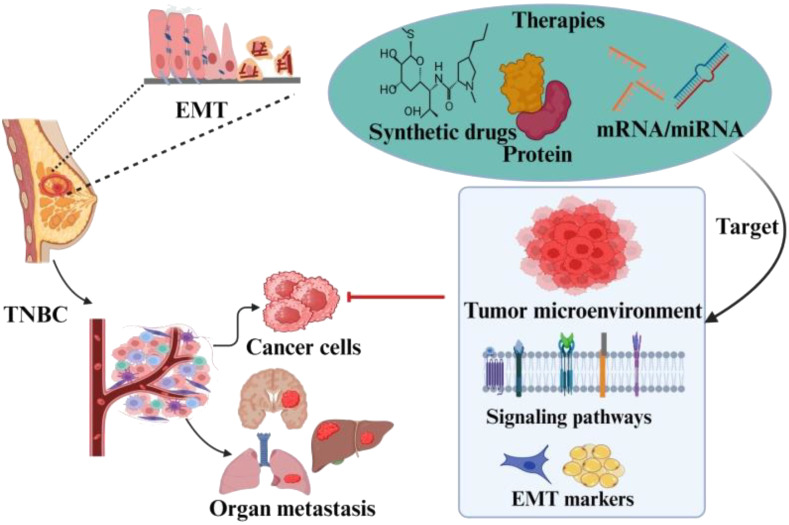

## 1 Introduction

Breast cancer (BCa) has become the second most prevalent cause of cancer related mortality among women, consistently increasing the incidence rate annually reported by the American Cancer Society (1, 2). It is the most commonly diagnosed cancer, with approximately 2.3 million cases worldwide annually (3). This unprecedented situation reminds us to investigate and develop improved therapeutic strategies thoroughly. Multiple factors, namely genetic, hormonal, and environmental, as well as other factors associated with lifestyle, are also involved in BCa pathogenesis. Therefore, BCa patients present various clinical, pathological, and molecular abnormalities (4). The expression of estrogen receptors (ER), progesterone receptors (PR), and human epidermal growth factor receptor 2 (HER-2) determines the variability in subtypes of BCa, including luminal A/B, HER-2 overexpression and triple-negative breast cancer (TNBC) (5). TNBC is a subtype, accounting for around 15-20% of all BCa cases, and is characterized by the absence of ER, PR, and HER-2 (6, 7). TNBC is further sub-classified as basal-like, luminal, and mesenchymal based on gene expression profile (8). It is the most heterogeneous among all BCa subtypes and referred as basal A and basal B type. Triple-negative A (basal A) cells are called basal-like as they are enriched with basal markers, including cytokeratins. Phenotypically, triple negative A type cells, highly differentiated subtypes within TNBC subtypes, can exhibit either luminal-like or basal-like morphologies. Thus, triple negative A subtypes closely mimic the core basal tumor subtype. On the other hand, Triple-negative B type (basal B), represents the mesenchymal cluster or normal-like/claudin-low, over-expressed genes and are associated with tumor invasiveness and aggressiveness (9). These subtypes can be utilized for modeling claudin-low or metastatic breast cancer due to their abundance of epithelial-mesenchymal transition (EMT) and stem-cell markers (9).

**Figure 1:**
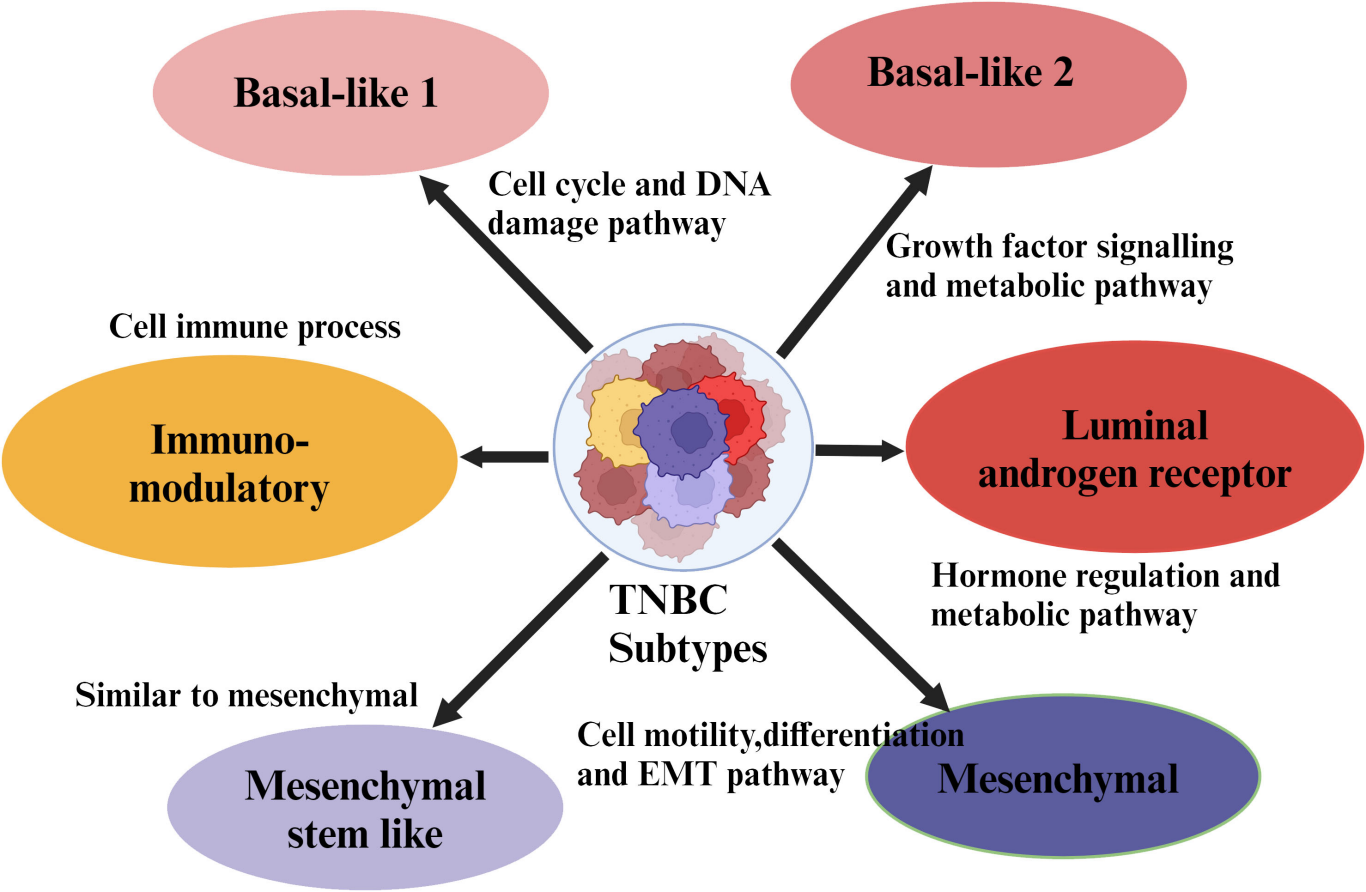
Molecular subtypes of triple negative breast cancer. Major subtypes of breast cancer such as luminal A, luminal B, HER-2 enriched, and TNBC are classified based on the expression of receptors on their cell surface. Further, the TNBC subtypes are sub-classified based on the specific site of the tumor.

In comparison to other subtypes, TNBC frequently occurs in young women and is characterized by enormous aggressiveness and mortality rates (10, 11). Around 45% of patients diagnosed with TNBC exhibit distant metastases in the brain or other parts, and the average survival rate ranges from 13.3 to 18 months (12). Several studies have demonstrated that approximately 25% of individuals diagnosed with TNBC can survive for 5 years or more (13). Conventional chemotherapy has shown significant efficacy against TNBC patients. However, its adverse effects become a potential threat, and some patients fail to get any clinical advantages from this treatment. Thus, identifying specific targets for TNBC therapy is a challenging and crucial therapeutic issue that needs to be resolved (14–19). Exploiting whole genome sequencing has provided significant heterogeneity in TNBC and played a critical role in categorizing several subtypes within the TNBC population (20). Different subtypes of TNBC are presented in **Figure 1**. Due to growing development and advances in bioinformatics tools, the study is gradually moving toward large samples, multi-omics, and refinement of complex data. Potential therapeutic targets derived from genomes, transcriptomics, metabolomics, and proteomics have recently emerged, and many of these research findings have great clinical translational significance and gained enormous importance (21).

### 1.1 Epithelial-mesenchymal transition

**Figure 2:**
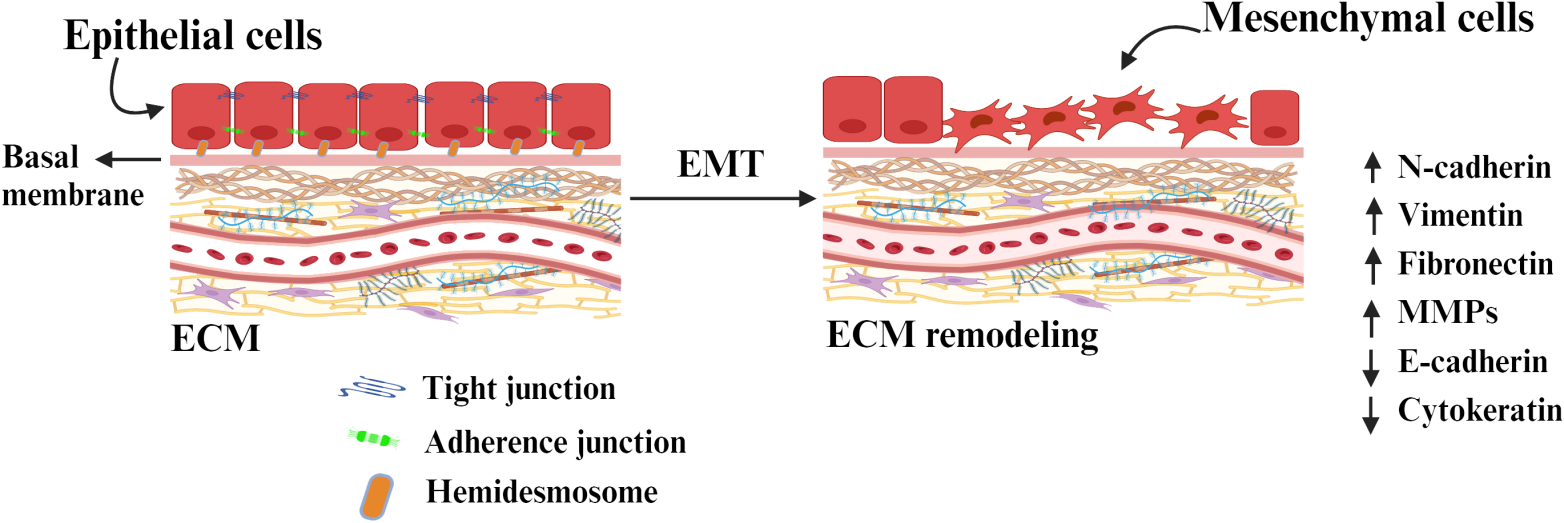
The conversion of epithelial cells to mesenchymal cells through epithelial-mesenchymal transition (EMT) by detaching from the basement membrane. Tight junctions tightly hold together the basal and apical parts of the epithelial cells. The cells are connected to the ECM through adherence junction and hemidesmosome. The epithelial state of the cells is maintained through the expression of a molecule associated with it and retains its polarity. The expression of EMT-inducing molecules and transcription factors remodel the ECM components and induces EMT.

The biological process by which the polarized epithelial cells transform into mesenchymal cell phenotype is called Epithelial to mesenchymal transition (EMT). The significant structural change in extracellular matrix (ECM) induces proliferation, migration, adhesion, invasion, and EMT (22). EMT defines the biological process by which epithelial cells lose their adhesion properties and gain migratory and invasive properties (mesenchymal characteristics), crucial in initiating tumor cell migration and metastasis development (23). This process involves various biochemical changes that result in increased migratory capacity, invasiveness, resistance to apoptosis, and significantly increased production of extracellular matrix (ECM) components (24). Several molecular mechanisms are involved in initiating and facilitating the completion of an EMT (25). These mechanisms involve the activation of transcription factors, expression of particular cell surface proteins, reorganization and expression of cytoskeletal proteins, production of enzymes that degrade ECM, and alterations in the expression of specific microRNA (miRNAs). In numerous instances, the factors involved in the process are utilized as biomarkers to exhibit the progression of a cell undergoing an EMT (**Figure 2**).

### 1.2 EMT in TNBC invasion and metastasis

Invasion and metastasis are characteristic features of tumor cells and occur due to transition in intrinsic properties, particularly associated with tumor microenvironment (26). During the progression of a tumor, EMT plays a crucial role and is considered a critical factor in the development and metastasis of TNBC (27). Carcinoma cells in the primary tumor lose cell-cell adhesion due to E-cadherin repression and gain enhanced invasive properties, allowing them to break through the basement membrane and enter the bloodstream via intravasation. Once the tumor approaches a new metastatic site, it may undergo other processes to enhance growth. TGF-β plays a vital role in regulating the morphogenesis and proliferation of normal mammary epithelial cells. However, BCa cells show high resistance towards TGF-β and act as cancer development promoter, which, in turn, modulates angiogenesis, invasion, and resistance against therapeutic interventions (28). Activation of Ras-expressing mammary epithelial cells by TGF-β promotes EMT and inhibits apoptosis (29). It has been discovered that TGF-β plays a crucial role in inducing EMT by regulating through Smad and non-Smad signaling pathways (30). The study revealed that the NF-κB pathway activation leads to EMT induction in TNBC (31). Downregulation of E-cadherin as a result of EMT is indicative of TNBC development. Three distinct core families of transcription factors mediate this regulation. One of the core families belongs to the Snail zinc finger protein family comprises transcription factors Snail 1 and Slug. Another family engaged in regulation is the E-box binding zinc finger protein family, which consists of ZEB 1 and ZEB 2 transcription factors. The basic helix ring helix protein family, consisting of transcription factors TWIST 1, TWIST 2, and E12, is also implicated in the regulation of EMT (25). The overexpression of Snail has been observed in both epithelial and endothelial cells of invasive BCa in comparison to undetectable levels in normal breast tissue (32). The Snail has also been associated with high-grade tumors, metastasis, recurrence, and poor prognosis (33). In addition, the Snail family’s proteins work with other transcription factors to coordinate the combined regulation of EMT. Recent studies have shown a strong association between Slug and TWIST expression in BCa (34). The Snail and TWIST proteins promote ZEB1 expression during EMT (35).

**Figure 3:**
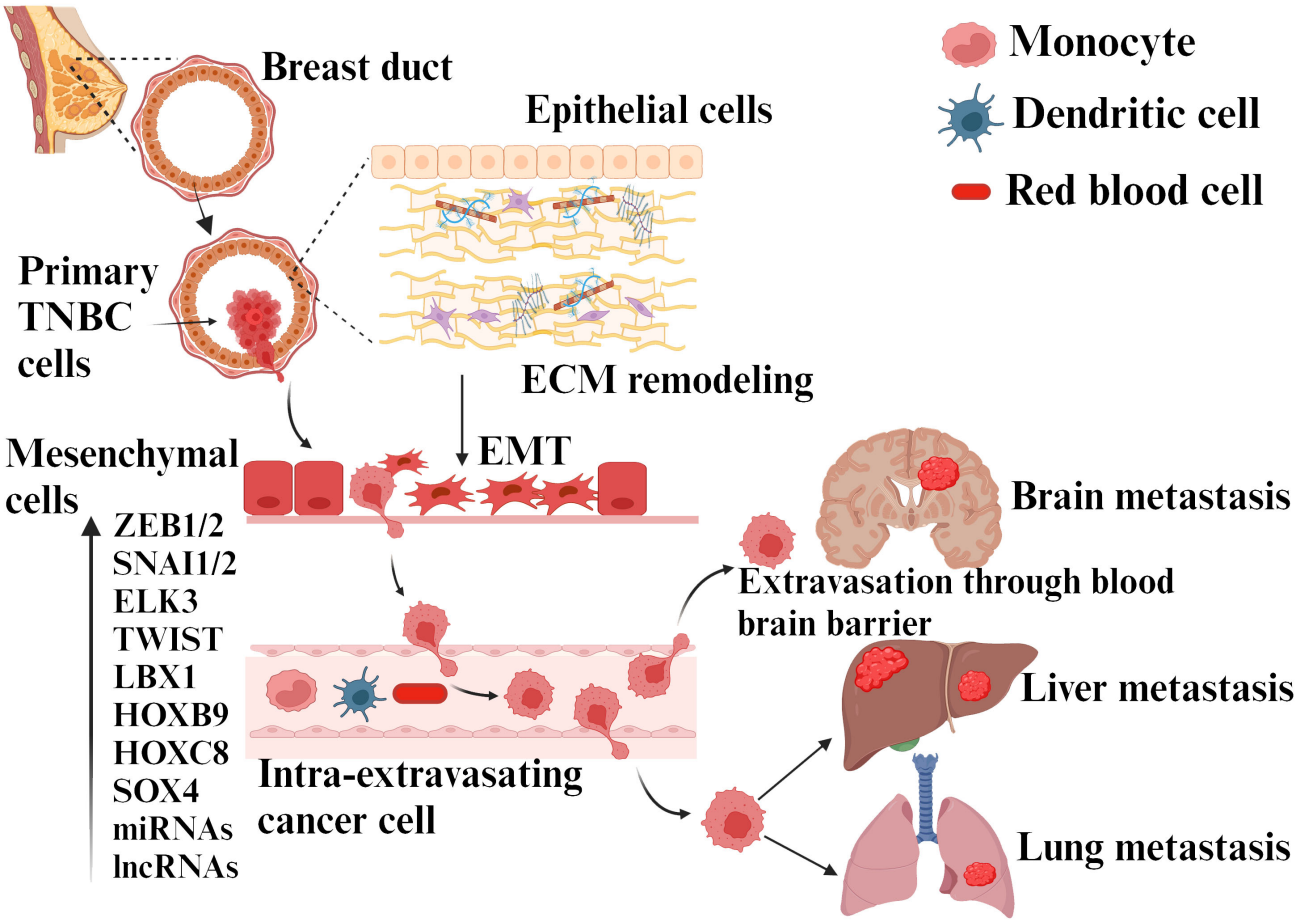
TNBC metastasis due to EMT transition. EMT is induced by various cytokines, molecules secreted by different cells present in the tumor microenvironment, ECM elements, and hypoxia. This process involves different genes, miRNAs/transcription factors, etc. The cancer cells achieve migration, invasion, intravasation, and progression to distant sites by activating EMT and mesenchymal phenotype upon ECM remodeling.

Additionally, ZEB1 has been shown to exhibit stem cell-like properties in TNBC (36). This causes significant risk to cancer patients, as EMT not only allows tumor cells to enter the bloodstream but also enhances their stemness, increasing their potential for tumorigenesis and proliferation (37). The role of EMT and associated factors in the initiation and metastasis of TNBC is presented in **Figure 3**.

Recent studies also indicate that miRNAs are critical regulators of EMT. miRNAs are short, non-coding, single-stranded RNAs of 20–22 nucleotides that regulate gene expression at the post-transcriptional level (38). The miRNAs are frequently dysregulated in human malignancies and have been linked to the regulation of several cellular processes. According to several reports, specific miRNAs directly target mRNAs of ZEB1 and ZEB2 in cancer cells by upregulating E- cadherin and reducing cell motility (39).

The Protein kinase C iota (PKC-ι) has been also observed to promote TNBC invasion during EMT as well (40). Inhibition of PKC-ι increases the level of E-cadherin and RhoA, while simultaneously decreases vimentin and Par6 (partitioning defective 6 homologs) (41, 42). The role of EMT transcription factors, including SNAIL, TWIST, and ZEB, in BCa invasion mediated via adherens junction has been revealed from several findings (43). Therefore, the effects of EMT and its association with invasion and metastasis appear to be highly dependent on the specific environment. Mesenchymal-to-epithelial transition (MET) is the reversible process of transforming EMT to MET derivatives. It is considered a hallmark of phenotypic plasticity, and the exact mechanism is still poorly understood. However, the MET associated with kidney formation is the most extensively studied example, and it is driven by genes such as bone morphogenetic protein 7 (Bmp7), paired box 2 (Pax2), and Wilms tumor 1 (Wt1) (44). During the MET process, genes associated with epithelium are up-regulated, while mesenchymal-associated genes are downregulated through specific signaling pathways (45).

In addition to these factors related to tumor microenvironments, autophagy has also a role in regulating EMT by activating energy response pathways, initiating EMT-inducing signaling pathways, and managing the breakdown of EMT-related adhesion and cytoskeletal components, as well as EMT-TFs (46). Recent studies have also revealed a shift in the primary effects of EMT, moving away from invasion and metastasis, towards resistance against chemotherapeutic agents.

### 1.3 TNBC treatment

TNBC has demonstrated sensitivity towards chemotherapy, making it a current standard of care (SOC), though it has limited advantages (47). The Food and Drug Administration (FDA) approved anti-metabolites, paclitaxel, anthracyclines, and neoadjuvant chemotherapy regimens for patients with TNBC (48) presented in **Table 1**. Patients with TNBC have demonstrated some efficacy with conventional treatment. However, the toxicity of chemotherapy on individuals is a concern, and some may not even experience any therapeutic improvement. Thus, identifying appropriate targets for precise TNBC therapy is a difficult and crucial therapeutic issue that needs to be resolved. Chemotherapeutic drugs such as fluorouracil (5-FU), doxorubicin, and cyclophosphamide have been used for TNBC treatment (49). The current SOC for individuals diagnosed with early TNBC is administered neoadjuvant chemotherapy, along with surgical intervention (50). Currently, no universally accepted chemotherapy treatment option is available for individuals diagnosed with recurrent or resistant TNBC (51). The length of treatment responses is typically short, often followed by rapid relapse, and the occurrence of metastases in visceral organs and the brain is prevalent (52). New therapeutic alternatives have recently become available for patients with advanced TNBC, mainly when surgery is not an option. The therapeutic options currently accessible for individuals diagnosed with advanced TNBC include capecitabine and gemcitabine, eribulin, and DNA cross-linker platinums such as cisplatin and carboplatin (53–55). TNBC has more immunogenic properties than other BCa subtypes with tumor-infiltrating lymphocytes (TILs) in its microenvironment. However, it also exhibits a significant upregulation of programmed cell death ligand 1 (PDL-1) (56, 57). Therefore, the use of immunochemotherapy has been established as a SOC for patients diagnosed with PDL-1 positive, unresectable, locally progressed, or metastatic TNBC.

**Table 1:** List of drugs administered to TNBC patients.

Drugs	Target	Action Mechanism	Limitations	References
5-fluorouracil	Thymidylate synthase inhibitor	blocks the action of thymidylate synthase which in turn inhibits the synthesis of pyrimidine thymidylate thus stopping DNA production and replication	aberrant activation of different signaling pathways, and resistance to DNA damage	(58, 59)
Doxorubicin, Daunorubicin, Epirubicin, Idarubicin	Topoisomerase II inhibitors	acts mainly via intercalating with DNA and interfering with DNA metabolism and RNA production	Doxorubicin-resistant TNBC cells exhibit rapid growth with CSCs and EMT Phenotype	(60, 61)
Cyclophosphamide	T-regulatory cell elimination	interfere with CD4 + 25+ TREGs and restores T and NK effector functions	demonstrates a significantly increased risk of premature menopause	(62, 63)
Capecitabine	Thymidylate synthase inhibitor	inhibits the synthesis of thymidine monophosphate which is required for the DNA synthesis	dihydropyrimidine dehydrogenase (DPD) decreases the activity and makes it resistant	(64)
Gemcitabine	Orthohepevirus A replication inhibitor	blocks the synthesis of new DNA by incorporating gemcitabine di and triphosphate into DNA resulting in cell death	decreased expression of (hENT1) and increase of glycolysis is strongly associated with gemcitabine resistance in TNBC	(65)
Eribulin	Microtubule inhibitor	eribulin disrupts the formation of the mitotic spindle by inhibiting microtubule polymerization and prevents cell division	cause changes in heart rhythms, such as a condition called QT prolongation	(66, 67)
Cisplatin Carboplatin	Binds to and cross-links DNA,	damages the DNA and also interferes with the DNA damage repair system	negative regulation of apoptosis makes it resistant	(68–70)
Pembrolizumab Atezolizumab,	PDL1 or PD1 inhibitor	Activates antitumor immunity by blocking the interaction of PD-L1 with programmed cell death protein 1 (PD-1) and CD80 receptors	does not improve prognosis in early relapsing	(71, 72)
Olaparib Talazoparib	PARP inhibitor	blocks PARP enzyme that is involved in DNA repair and affects the DNA damage response pathway	causes gastrointestinal-related ailments	(73, 74)
Paclitaxel	Mitotic inhibitor	stabilizes the microtubule polymerization and does not allow depolymerization during cell division	BCL2-mediated drug resistance	(75, 76)
Geftinib	Tyrosine kinase inhibitor	inhibits intracellular phosphorylation of tyrosine kinases associated with transmembrane cell surface receptors and induces growth inhibition and cell death	QT prolongation	(77)
Rapamycin	mTOR kinase inhibitor	inhibits the mTOR enzyme controls many cellular processes, including metabolism, autophagy, and survival in TNBC	rapamycin has a limited ability to regulate all actions of mTORC1	(78)
Dasatinib	Abl/Src inhibitor	blocks the action of an abnormal protein that signals cancer cells to multiply and reduce tumor growth, invasion, and recurrence	potential pulmonary toxicities such as pulmonary arterial hypertension and pleural effusion, limit its clinical use	(79)
Bicalutamide, Enzalutamide	Anti-AR therapy	blocks the activity of androgens of adrenal and testicular origin which stimulate the growth of malignant tissue	responsible for the reproductive system and breast disorders	(80)

In 2017, pembrolizumab was approved as a histological agonist immunotherapy against tumors involving microsatellite instability and mismatch repair deficiency (81, 82). The FDA approved atezolizumab combined with nanoparticle albumin-bound (nab)-paclitaxel as a first-line therapy against TNBC (83). Tumors with the absence of BRCA1 and BRCA2 show impaired homologous recombination repair and are sensitive to poly(ADP-ribose) polymerase (PARP) inhibitors (84, 85). In 2018, the FDA approved olaparib and talazoparib for treating advanced stage HER2 negative BCa patients with BRCA1/2 mutation. Olaparib was approved upon the significant activity noticed compared to capecitabine or eribulin as chosen by the physician (86). Olaparib demonstrated substantial improvement in median progression-free survival (PFS) in comparison to the control group, with a notable increase of 42% (7 vs. 4 months) (87). Olaparib demonstrated a promising safety profile during prolonged exposure, with no indication of cumulative toxicity. In patients with locally advanced or metastatic BCa with germline BRCA mutation who have previously undergone chemotherapy, talazoparib has been found to increase the average PFS by 46%, with a duration of 8.6 months compared to 5.6 months (88). Another SOC neoadjuvant therapy drug, paclitaxel, for the treatment of patients with TNBC, shows limited benefit for locally advanced or metastatic disease (89). The use of lapatinib and geftinib in combination with capecitabine was approved by FDA as a combination therapy for breast cancer patients (78). The other drug, rapamycin, has been used to target AKT/mTOR pathway and inhibit proliferation of tnbc.

## 2 Targeted therapy associated with EMT

TNBC is a diverse disease with various genetic variations and molecular subtypes. Due to factors like numerous metastases, extensive organ metastases, and too close to large blood arteries, only a few patients with TNBC metastases are suitable for surgical treatment. To provide patients with personalized therapy and increase their survival chances, investigators studying the metastatic process must bring innovative therapies for patients with advanced BCa. Targeted therapy is one of the critical current options with enhanced efficacy and less adverse effects on patients. The role of different potential targets in EMT is discussed further.

### 2.1 EMT and genes

Molecular biomarkers are now being studied as possible treatment targets. It has been observed that 11-20% of individuals diagnosed with TNBC, who have not been specifically selected based on their family history, had a hereditary mutation in the BRCA1 or BRCA2 (BRCA1/2) genes. Moreover, current research indicates that deleterious mutations in additional genes associated with cancer susceptibility are also linked to TNBC (90, 91). Other than BRCA1/2 detection, four other established genes (CDH1, PTEN, STK11, TP53) associated with BCa were identified and established as alternate options for diagnosis. The loss of CDH1 gene expression is strongly associated with BCa progression in patients (92). Another critical gene, PTEN expression loss, demonstrates poor prognosis and treatment response. STK11 is a tumor suppressor gene and loss‐of‐function mutations cause tumorigenesis in TNBC (93). Mutation in TP53 is observed in 18%–25% of primary BCa and roughly 80% of TNBCs, which is noticeably more frequent than other BCa subtypes (2, 17).

Later, panels were expanded to include an additional 15-20 candidate genes having similar function to BRCA1/2 in DNA double-strand break repair (ATM, BARD1, CHEK2, PALB2). Furthermore, 25–40 genes (including CDKN2A, MEN1, MLH1, MSH2, MSH6, and MUTYH) that cause cancer risk at different organ sites were identified and added to the existing panel. The number of genes on panels that may be linked to cancer has recently increased to over 100, yet many lack strong enough data to perform patient treatment (94). Before the clinical use of multiple genes panel testing, there was limited understanding of gene alterations in TNBC beyond BRCA1/2. A total of 122 DNA repair genes from germline DNA samples of BCa patients were sequenced, out of which 17 genes (ATM, BRCA1, BRCA2, BARD1, BRIP1, CHEK2, CDH1, MRE11A, NBN, PALB2, PTEN, RAD50, RAD51C, RAD51D, STK11, TP53, and XRCC2) are associated with increased risk of developing BCa were observed (**Table 2**). A prior report demonstrated that 271 deleterious mutations were detected among patients (95). In this finding, most of these changes (57% or 155 mutations) were observed in BRCA1 mutated tumors, while (18% or 49 mutations) were shown in BRCA2. The remaining (25% or 67 mutations) were distributed among additional susceptibility genes. Notably, PALB2 accounted for 7.7% of these mutations, followed by BARD1 (3.3%), RAD51D (2.5%), RAD50 (2.2%), and RAD51C (2.2%) (90). Of the TNBC patients, 3.7% had these probable harmful mutations in non-BRCA1/2 genes, while 11.2% had BRCA1/2 mutations. Interestingly, no CHEK2 mutations were discovered, which aligns with its link to hormone receptor-positive BCa (96).

**Table 2:** List of genes involved in EMT pathogenicity due to altered expression.

Genes	Alteration	Function	References
BRCA1/2	Mutations	CNS metastasis	(97, 98)
ATM	Upregulated	Stabilizes ZEB1 and promotes EMT and radioresistance	(99, 100)
BARD1	Upregulated	Associated with TNM staging and overexpressed in cytoplasm	(90, 101)
CHEK2	Mutations	CHEK2 mutation promotes lymph node metastasis	(102)
CDKN2A	Upregulated	Regulates EMT markers Snail1, Twist1, Zeb1, vimentin, MMP9, and E-cadherin	(103)
MEN1	Upregulated	More localized in the nucleus and promotes breast cancer	(104)
MLH1	Downregulated	The mismatch repair gene acts as a tumor suppressor	(99, 105)
MSH2 and MSH6	Upregulated	Mismatch repair genes form heterodimers and promote the BLBC subtype	(105)
MUTYH	Upregulated	MUTYH and BRAC1 play a synergistic role in metastasis	(106)
BRIP1	Mutations	DNA repair gene helps in the DNA repair function of BRCA1	(90)
CDH1	Upregulated	SND1 and DNMT3A lead to aberrant methylation patterns of CDH1 and promotes metastasis	(98, 107)
MRE11A	Upregulated	Missense mutation-driven alterations in the normal function of nuclease and DNA-binding activities promote breast cancer	(108)
PTEN	Mutation	EGF-induced chemotaxis of human breast cancer cells	(109)
RAD50, RAD51C, RAD51D	Downregulated/Mutations	Disruption of homologous recombination repair due to mutation promotes TNBC	(110)
TP53	Mutations	Stabilization of mutant P53 enhances HSP90 and promotes metastasis	(111)
STK11	Mutations/ Downregulated	The tumor suppressor gene regulates tumor invasive and metastasis potential through MMP2, MMP9, and VEGF EMT markers	(112)
XRCC2	Mutations	Lymph node metastasis	(113, 114)

### 2.2 EMT and miRNA

Several studies have evidenced the correlation between different miRNAs and underlying mechanisms of disease progression in TNBC (115–125). These mechanisms involve many processes, such as EMT, cellular migration, invasion, and metastasis (96, 126). Numerous studies suggest that miRNAs have a role in the EMT mechanism. The miR-200 family, which includes miR-200a, miR-200b, miR-200c, miR-141, and miR-429, is thought to contain the majority of miRNAs that negatively regulate EMT. The miR-200a is an EMT inhibitor that targets the E-cadherin repressor ZEB1/2 to maintain the epithelial phenotype (127).

Furthermore, miR-200b inhibits FUT4 expression in BCa cell lines, inhibiting TNBC migration and metastasis via inactivation of EGFR and downstream PI3K/Akt signaling cascade (128). In the meantime, TNBC’s epithelial phenotype is maintained by blocking the genes involved in cell motility, reducing cell growth, and encouraging apoptosis (129). Due to these properties, the miR-200 family is currently considered one of the promising therapeutic targets for the treatment of TNBC (130).

On the other hand, several other miRNAs play significant roles in increasing migration, invasion, and metastasis (131). The upregulation of miR-21 facilitates the proliferation of cancer cells (132). Moreover, 3’ UTR of LZTFL1 (leucine zipper transcription factor-like 1) along with miR-21 activates proliferation and metastasis (133). The upregulation of miR-21 causes increased cell invasion and proliferation in TNBC cells (MDA-MB-468). However, the PTEN gene was observed to be downregulated. This finding highlights the significance of the suppression of miR-21 and the overexpression of PTEN as a prospective therapeutic approach to predicting and assessing individuals diagnosed with TNBC (134). An overview of specific miRNAs linked to the pathophysiology of TNBC and their established targets and associated roles is presented in **Table 3**.

**Table 3:** Abberated expression of some miRNAs in TNBC and their functions.

miRNA	Change in expression	Targets	Function	References
miR-9	Upregulated	CHN1	• Inhibition of EMT • Worse disease-free survival due to high expression	(122)
miR-10b	Upregulated	HOXD10	• Cell migration and invasion induction • Lymph node metastasis due to high expression	(120)
miR-21	Upregulated	PDCD4, PTEN, H1F1α	• Induction of cell proliferation and invasion • Enhanced expression causes poor prognosis	(117)
miR-29	Upregulated	TTP	• Metastasis activation • Inversely correlated with stemness and no association with prognosis	(118)
miR-182	Upregulated	PFN1, FOXF2	• Induction of cell proliferation and invasion	(125)
miR-221	Upregulated	CDH1	• Induction of breast cancer progression	(123)
miR-145	Downregulated	MMP11, Rab27a	• Cell invasion inhibition through post-transcriptional regulation of target genes	(124)
miR-200	Downregulated	ZEB1/2, TWIST, CDH1, EPHA2	• Cell migration and invasion inhibition • Promotes differentiation of undifferentiated epithelial cell line • Associated with chemoresistance	(130)
miR-199a-5p	Downregulated	ZEB1, CDH1, TWIST	• Inhibition of EMT, migration, invasion, and tumor growth	(115)
miR-206	Downregulated	TM4SF1, CORO1C	• Suppression of tumor proliferation, migration, and invasion	(119)
miR211-5p	Downregulated	SETBP1	• Inhibition of proliferation, invasion, migration, and metastasis	(116)
miR-361-5p	Downregulated	RQCD1	• Inhibition of tumor migration and invasion	(121)
miR-603	Downregulated	eEF2K	• Suppression of cell proliferation and invasion	(135)
miR-4306	Downregulated	Cdc42, VEGFA	• Repression of cell proliferation, migration, and invasion	(136)

CHN1, Chimerin 1; HOXD10, Homeobox D10; PDCD4, Programmed Cell Death 4; PTEN, Phosphatase and tensin homolog; H1F1α, hypoxia-inducible factor-1-alpha; TTP, Thrombotic thrombocytopenic purpura; PFN1, Profilin-1; FOXF2, Forkhead box protein F2; CDH1, Cadherin-1; MMP11, Matrix metalloproteinase 11; Rab27a, Ras-related protein; ZEB1, Zinc finger E-box-binding homeobox 1; TM4SF1, transmembrane 4 L six family member 1; SETBP1, SET Binding Protein 1; eEF2K, Eukaryotic elongation factor-2 kinase; Cdc42, Cell division control protein 42; VEGFA, Vascular endothelial growth factor A.

### 2.3 EMT and lncRNA

Non-coding RNAs (ncRNAs) are classified into two groups based on their transcript size. Typically, ncRNAs are comprised of less than 200 nucleotides. These ncRNAs include miRNAs, piwi-interacting RNAs, small nucleolar RNAs (snoRNAs), and short interfering RNAs (siRNA). The second group is long non-coding RNAs (lncRNAs) containing more than 200 nucleotides. lncRNAs include intergenic, intronic, sense, antisense, enhance, and bidirectional (134). The involvement of lncRNAs in several biological processes, including apoptosis, cellular proliferation, cellular differentiation, metastasis, and chromatin remodeling, has been identified. Many tumors, including TNBC, have exhibited abnormal expression patterns of several lncRNAs (137). LncRNAs can bind and compete with miRNA-bound mRNA, resulting in altered regulation of miRNA-mediated genes. They constitute an endogenous RNA network (ceRNA) involving mRNAs and lncRNAs as a post-transcriptional regulatory network in TNBC (138). lncRNA HOTAIR, a well-investigated lncRNA in cancer, has demonstrated its ability to induce alterations in chromatin structure and gene expression, hence facilitating the process of invasion and metastasis, specifically in BCa. Several other lncRNAs have recently been linked to TNBC-related ECM/EMT molecules. The expression of cytoplasmic lncRNA, namely LINK-A (long intergenic non-coding RNA for kinase activation), is involved in growth factor-dependent phosphorylation, stability, and activation of HIF1α and linked with TNBC (139). A summary of different lncRNA and their functions in TNBC is documented in **Table 4**.

**Table 4:** LncRNA and associated target genes of TNBC.

LncRNA	Associated genes	Function	References
HAS2-AS1	HAS2	Abnormal HA accumulation causes cell dedifferentiation, proliferation, and migration.	(140)
HOTAIR	HOXA9, PTEN, AR	Abnormal regulation of apoptosis, the cell cycle, EMT, autophagy, self-renewal, and metabolism	(141)
LINK-A	HIF1α, EGFR	Promotes breast cancer glycolysis reprogramming and tumorigenesis	(139)
SNHG12	MMP13	Evade immune-mediated attack and enhance the polarization of effector immune cells	(142)
SKAI1BC	KAI1	Suppresses the KAI1/CD82 metastasis-suppressing gene	(143)
PVT1	KLF5, β-catenin	PVT1 enhances the resistance of the TNBC to doxorubicin	(144)
LNC01638	metatherian TWIST,	Induce MTDH-Twist1 signaling by inhibiting degradation of SPOP-mediated c-Myc in TNBC	(145)
MIR100HG	CDK 18, WEE1, CCNF, CDKN1B, CDC25A	Promote the proliferation of TNBC and increase the proportion of cells in the S phase	(146)
AWPPH	FZD7	Regulate cancer cell proliferation and chemosensitivity in TNBC	(132)
POU3F3	Cas-9	Regulates proliferation and apoptosis in TNBC through caspase 9	(147)
ZEB2-AS1	ZEB2	Promotes proliferation, metastasis, and EMT in TNBC	(148)
MIR503HG	MMP9, Olfactomedin 4	Induce cell proliferation, invasion, metastasis, apoptosis, angiogenesis	(149)

HAS2, Hyaluronan synthase 2; HOTAIR, HOX transcript antisense RNA; AR, Androgen receptor; LINK-A, Long intergenic non-coding RNA for kinase activation; EGFR, Epidermal growth factor receptor; SNHG12, Small nucleolar RNA host gene; KLF5, Krueppel-like factor 5 FZD7, frizzled homolog; Cas-9, CRISPR associated protein 9.

The snoRNA host gene 12 (SNHG12), a transcriptional target of c-myc, is highly increased in TNBC. SNHG12 may enhance cell motility through modulating MMP 13 expression (150). Another lncRNA is described as a suppressor of KAI1 in BCa (SKAI1BC) that suppresses the KAI1/CD82 metastasis suppressor gene and promotes TNBC (151). It has been demonstrated that two lncRNAs, Airn and PVT1, control TNBC carcinogenesis by acting opposite to each other on the β-catenin signaling pathway (131). Through TWIST 1 expression, another lncRNA, LINC01638, preserves the ϵμT characteristics of TNBC cells. The tumor growth and metastasis are inhibited through the knockdown of LINC01638 and MIR100HG, which act as oncogenes via controlling p27 (152). The lncRNA AWPPH has been implicated in promoting TNBC growth through the upregulation of frizzled homolog 7 (FZD7) (153) and/or its interaction with miRNA-21 (132). The level of lncRNA POU3F3 in TNBC patient’s plasma has been found to increase as compared to those of normal individuals, and a negative correlation between lncRNA POU3F3 levels and cleaved Caspase 9 was observed. This means that when the level of POU3F3 increases, then caspase 9 decreases, promoting cell proliferation and inhibiting apoptosis in TNBC (147). Many studies have demonstrated that lncRNAs such as HCP, PAPAS, and LUCAT1 have a role in promoting TNBC through the modulation of specific miRNAs (miR-219a-5p, miR-34a, and miR-5702, respectively) (154). The increase of LncRNA-ZEB2-AS1 showed enhanced proliferation and metastasis due to the upregulation of ZEB2 of MDA-MB-231 cells in SCID mice (90). LINC01638 has been found to inhibit the degradation of c-Myc and increase TWIST 1 expression, thereby inducing EMT (145). The lncRNA DLX6-AS1 exhibits an increased expression of EMT markers, promoting cell survival, and enhances resistance to the chemotherapy drug cisplatin in TNBC cells by regulating miR-199b-5p/PXN (155). The inhibition of cell proliferation, invasion, and migration, as well as the enhancement of apoptosis and regulation of the cell cycle, were seen upon the action of lncRNA, RMST (rhabdomyosarcoma 2-associated transcript) (156). The overexpression of another lncRNA, for example, NEF, has been observed to prevent the migration and invasion of TNBC cells (157). The expression of lncRNA PTCSC3 is decreased, but lncRNAH19 has shown increased expression and established an inverse relationship with PTCSC3 levels in TNBC patients. The overexpression of PTCSC3 results in the downregulation of lncRNA H19 in TNBC cells (158). Patients with TNBC who exhibit low expression of lncRNA MIR503HG have shown significantly poorer prognosis than those with high expression. It has also been observed that MIR503HG inhibits the migration and invasion of cells in TNBC by altering the miR-103/OLFM4 axis (159). The LncRNA TCONS_l2_00002973 positively correlates with lower-grade tumors and better survival outcomes. Additionally, it demonstrates inhibitory effects on cancer cell proliferation and promotes apoptosis, specifically in TNBC (160). The *in vitro* and *in vivo* studies have shown the inhibitory effects of lncRNA XIST on cell proliferation and EMT in TNBC cells by interfering with the activity of miR-454 (161).

### 2.4 EMT and proteins

The ECM proteins have a significant role in the tumor microenvironment (**Table 5**). Many ECM proteins include (BGN, CD44, CD109, DAG1, DCN, ECM1, EFEMP1, FMOD, IGFBP4, IGFBP7, LTBP1, L1CAM, LGALS1, LGALS3BP, LOXL2, LTBP1, NRCAM, P4HB, PLOD1, PPIB, TGF-β I, THBS1, TLN1, and TNC) have found highly expressed in TNBC. However, DCN and TGF-βI are highly expressed in normal cells (162). CD44 is a transmembrane glycoprotein and is highly expressed in TNBC. It plays a pivotal role in mediating cellular adhesion and signaling processes, and its presence in serum is considered a potential prognostic indicator in BCa (163). Recently, significant advancements have been made in developing nanoparticle drug delivery methods, especially targeting CD44 and CEA in colorectal cancer cells and TNBC (164). The insoluble form of CD109 is bound with TGF-β, which acts as a negative regulator and prevents TGF-β signaling (165). The high expression of CD109 in TNBC correlated with a higher histological grade and worse prognosis (166). ECM1, another protein present in the secretome of TNBC, is responsible for inducing angiogenesis and promoting tumor cell proliferation via EGFR signaling (167). Elevated expression of ECM1 in BCa is linked to poor prognosis (168). The glycoprotein Fibulin 3 (FBLN3), also known as EFEMP1, is a protein that interacts with ECM1 and is found in the secretome of TNBC (162). The overexpression of FBLN3 in BCa cases is characterized by low levels of HER2 expression, including TNBC (169). Additionally, it has been observed that FBLN3 has a role in enhancing the invasiveness of tumor cells in xenografts of TNBC (170). The function of FBLN3 in the TNBC secretome is still unknown. The TNBC secretome also contains the closely related protein Fibulin 1 (FBLN1), which interacts with fibronectin (171). The role of FBLN1 in estrogen signaling in BCa is demonstrated in numerous studies. FBLN1 expression is increased by estrogens, particularly that of the spliced variant FBLN1C (172). In an immunohistochemical study of BCa, FBLN1 expression showed an inverse correlation with cathepsin D (173). Interestingly, the TNBC secretome contained both cathepsin D and FBLN1 (174). Importantly, FBLN1 may be involved in BCa cell resistance to doxorubicin therapy (175).

**Table 5:** ECM proteins in secretome of TNBC.

Protein	Abbreviation	Function	References
Biglycan	BGN	Tumor angiogenesis, TME remodeling	(182)
Cluster of Differentiation 44	CD44	Suppress PD-L1 function	(183)
Cluster of Differentiation 109	CD109	Initiation, progression, and differentiation of BCa cells	(184)
Dystroglycan	DAG1	Involved in adhesion and wound repair of epithelium	(185)
Decorin	DCN	Wound repair, angiogenesis, metastasis	(186)
Extracellular matrix protein 1	ECM1	Maintenance of skin integrity and homeostasis	(187)
Fibulin 1	FBLN1	Confer resistance to doxorubicin on BCa treatment	(187)
Fibulin 3	FBLN3	Promote tumor cell invasiveness	(187)
Galectin 3 binding protein	LGALS3BP	Downregulates T-cell receptor expression	(188)
Lysyl oxidase like-2	LOXL2	Facilitate cell migration and the formation of metastases through inflammation in TME	(189)
Neuronal cell adhesion molecule	NCAM	Mediates adhesion, guidance, and differentiation	(190)
Protein disulfide isomerase, prolyl 4hydrxylase beta	P4HB	Promotes cancer progression via upregulation of EMT	(191)
Peptidyl-prolyl cis-trans isomerase B	PPIB	Promotes cell proliferation and invasion	(192)
Reelin	RELN	Promotes cisplatin resistance by inducing EMT	(193)
Tissue factor	TF	Promotes immune evasion by impeding T-cell infiltration	(194)
Talin 1	TLN1	Attenuates the migration of tumor cells by interfering with integrin β	(195)
Tenascin C	TNC	Modulates cell migration proliferation through induction of cytokines	(196)

Several insulin-like growth factor-binding proteins (IGFBPs) have been identified in the secretome of TNBC (176). Proteins belonging to this particular family can bind to IGF and subsequently enhance its half-life (177). The family member Cyr61 (alternatively referred to as CCN1 and IGFBP10) has shown expression in TNBC cells by interacting with the urokinase plasminogen activator receptor (uPAR) (178). It has long been demonstrated that Cyr61 stimulates angiogenesis and tumor development (179). High Cyr61 expression is associated with relapse in TNBC patients, and Cyr61 knockdown decreased TNBC cell line invasiveness, tumor burden, and microvascular density (178). Apart from these ECM proteins, the presence of α2,3-sialylated N-glycoproteins capable of binding lectins in the conditioned media of TNBC cell lines is also reported (180). The expression of polysialic acid in BCa is positively correlated with invasiveness and TNM staging in patients’ tumors. Furthermore, it has also been shown that the suppression of Sialyl transferase X (STX) results in decreased migratory capacity of MDA-MB-231 cells (181). These studies on identifying proteins inside ECM provide us with only a fraction of the overall story since many ECM proteins undergo post-translational modifications, which can also be a target.

## 3 EMT pathways regulators

**Figure 4:**
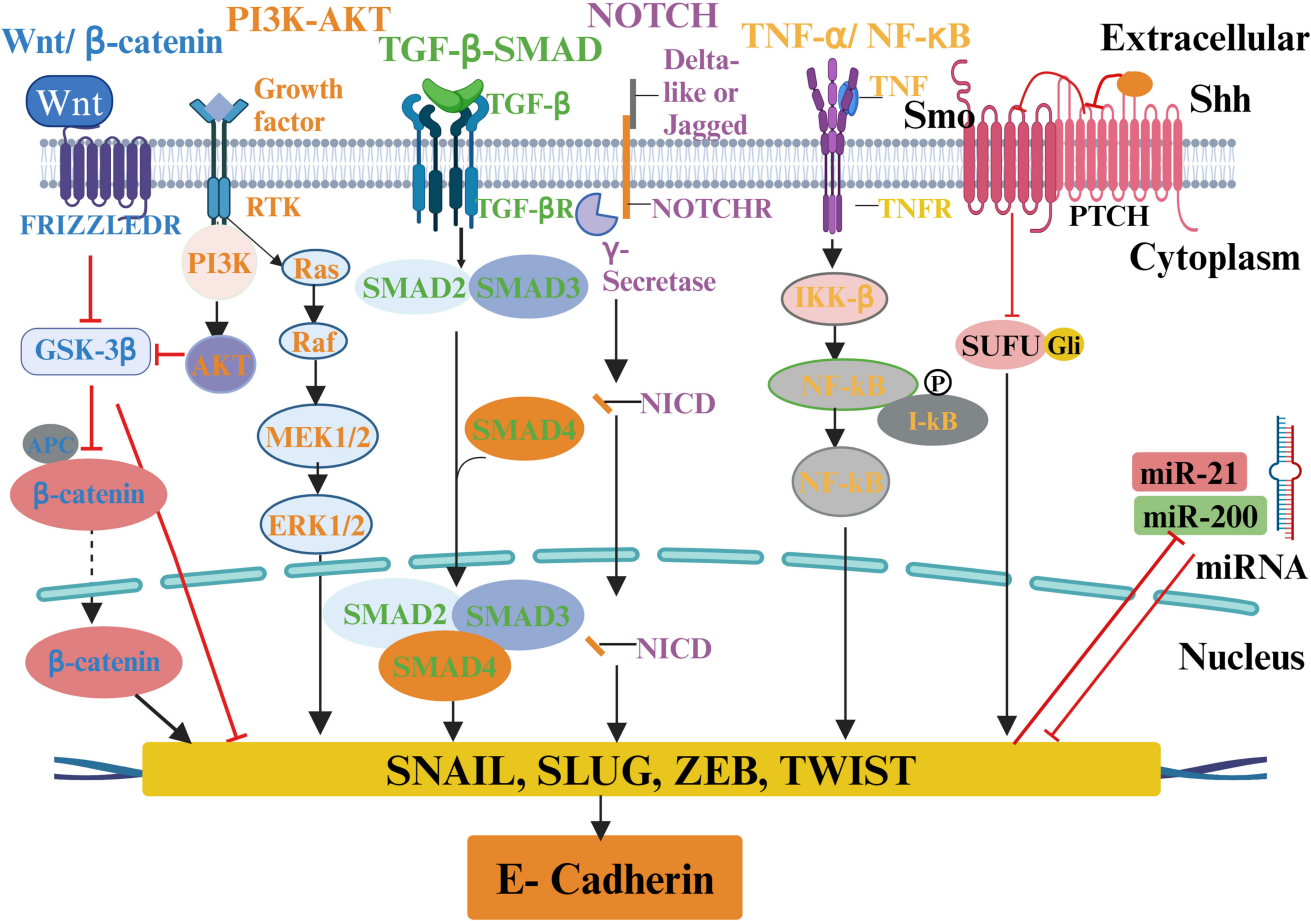
A schematic of various ECM signaling pathways involved in EMT regulation in tumor microenvironment. ECM molecules and proteins interact with the tumor microenvironment to activate multiple biochemical signaling pathways and regulate EMT transcription factors and different EMT inducers to cause EMT and tumor progression. The molecules involved in particular signaling pathways are presented with the same color.

Numerous signaling pathways, including TGF-β, Wnt/β-catenin, Notch, TNF-α/NF-κB, Hedgehog (Hh), and receptor tyrosine kinase (RTKs), are involved in EMT regulation (**Figure 4**) (197). The transcription factors Snail, ZEB1/2, and TWIST, along with miRNA, epigenetic regulators, and alternative splicing, are regulated by these pathways during cancer progression (198). Furthermore, it has been suggested that EMT facilitates the progression of early-stage primary tumors into invasive malignancies and contributes to the development of cancer cells exhibiting stem cell-like properties (36). These properties include enhanced self-renewal capacity, the ability to initiate tumor formation, and resistance to programmed cell death and chemotherapy (199).

### 3.1 TGF-β pathway in EMT

TGF-β is a critical cytokine that exhibits a significant role in initiating EMT programming (200). It plays a vital role in activating EMT and interaction with downstream signaling pathways during tumorigenesis (45). The dysregulation of TGF-β expression has been involved in the development of different types of cancer, including breast carcinogenesis (149). In EMT, intracellular Smad2/3 transducer proteins trigger the TGF-β signaling pathway (201). There are three types of TGF-β involved in Smad-dependent signaling: TGF-β1, TGF-β2, and TGF-β3, which are linked to three distinct receptor types: types I, II, and III. When TGF-β binds to TGF-βR-II, TGF-βR-I is activated, triggering the Smad2/3-dependent signaling pathways (202). TGF-β receptors activate Smad2/3, resulting in an active complex of Smad2/3 and Smad4 that regulates the altered expression of different genes associated with EMT (203). The studies found the suppression of invasiveness due to decreased expression of Smad2 and Smad3 (204). On the other hand, upregulation of Smad2 and Smad3 expression is associated with EMT (205). TGF-β activates the AKT/PI3K, Ras/Raf/MEK/ERK, and Wnt/β-catenin signaling pathways, which produce epithelial proteins in non-Smad signaling pathways (206). The regulation of transcription factors, including Snail, Slug, ZEB1/2, and TWIST, is mediated through Smad-dependent and Non-Smad pathways (207). The interaction of TGF-β with several signaling pathways, such as Notch, Wnt/β-catenin, nuclear factor NF-κB, and RTKs, results in the induction of EMT and is essential for preserving the mesenchymal characteristic of invasive and metastatic tumor cells (208). TGF-β signaling during EMT modifies the tight junction formation and triggers the activation of additional signaling pathways, including Wnt, Notch, and Hh MAPK pathways. TGF-β regulates various gene expressions, including core transcription factors Twist (TWIST 1 and TWIST 2), SNAI (SNAI 1 and SNAI 2), ZEB (ZEB 1 and ZEB 2), and Six family of homeobox (Six1) (209). The transcription of E-cadherin, occludin, and claudin is also regulated by TGF-β (208). TGF-β overexpression in BCa is linked to increased EMT (197). Its relationship to BCa stem cells in EMT has recently been discovered (210).

### 3.2 The Wnt/β-catenin pathway in EMT

The Wnt/β-catenin pathway is essential in EMT regulation in BCa. Many studies have shown the involvement of Wnt signaling in BCa metastasis, immune microenvironment, stemness maintenance, and resistance to therapies (211). The Wnt signaling pathway is controlled by either canonically (β-catenin-dependent expression) or non-canonically (β-catenin-independent expression) (212). The studies revealed that accumulation of β-catenin in the nucleus is the reason for the poor prognosis of BCa (213). The role of glycogen synthase kinase-3 beta (GSK-3β) in regulating β-catenin expression has been elucidated. The increase in the phosphorylation of GSK3β causes the degradation of β-catenin and regulates the Wnt signaling pathway (214). This pathway can regulate the expression of Snail and β-catenin, facilitating EMT and promoting metastasis through inhibition of GSK-3β activity (215). The elevation of SNAI1 expression activates Wnt/β-catenin, resulting in downregulation of E-cadherin and overexpression of vimentin within BCa cells (216). According to reports, the development of several types of BCa is associated with aberrant expression and sub-cellular localization of β-catenin correlated to activation of the Wnt signaling system (217). Even though the Wnt pathway is thought to be linked to the EMT in BCa, β-catenin is not enough to cause EMT on its own (218). β-catenin acts as a molecular bridge in tight junctions of epithelial cells and promotes cell-cell adhesion (219). The process of EMT involves the stabilization of β-catenin and the activation of the Wnt signaling pathway. This activation is closely associated with the involvement of a transcription factor known as T-cell factor/lymphoid enhancer factor (TCF/LEF) and several other components (220).

### 3.3 Notch pathway in EMT

The Notch pathway is involved in regulation and cell survival during cell development. It plays a vital role in the initiation and progression of cancer (221). Four Notch receptors and five ligands have been reported (222). Unusual or deviating from the norm. The association between Notch signaling and the TNBC subtype has been well established (223). The over-expression of the Notch receptor is associated with the aggressive, metastatic, and therapy-resistant phenotype that is the hallmark of TNBC. Deregulation of the Notch pathway with notch 1, 2, 3, and 4 receptors and ligands (Jagged1, 2 and Delta-like1, 3, 4) are known to be involved in the induction of breast cancer mesenchymal phenotype via interacting with RTKs, MAPK and PI3K signaling (224). The canonical Notch pathway operates through the interaction of the two important ligands delta and Jagged, creating two rounds of dissociation of the Notch receptor at the S2 point (225). The first cleavage is mediated by ADAM10 or ADAM17, followed by the second with γ-secretase generating Notch intracellular domain (NICD) that induces slug-mediated EMT (226) as outlined in **Figure 4**. The activation of the Notch signaling system induces the NF-κB pathway and regulates TGF-β involved in EMT programming. NUMB is an essential gene that mediates Notch signaling. It has been identified as a suppressor of EMT in human epithelial cells and TNBC cells (225). The downregulation of NUMB has been correlated with the increase in EMT (227). The association between the upregulation of Notch signaling and the overall survival rate of TNBC patients has already been demonstrated (228). The expression of Snail is regulated by Notch signaling via transcriptionally activating either Snail or lysyl oxidase (LOX) (229). Several studies revealed an association between Notch activation and hypoxia. One of the critical factors regulating tumor metastasis is hypoxia. Notch is a crucial bridge connecting the hypoxia response to EMT (230). Notch signaling increases the expression of LOX by triggering a hypoxia-inducible factor 1-α (HIF-1α). This, in turn, stabilizes Snail and leads to the upregulation of EMT programming, which induces the invasion of cancer cells (231). Notch 1- triggers the EMT process in TNBC (228, 232). Notch 2 was found to be involved in TNBC (228). Notch 3 is well known for its anti-metastatic or inhibitor of the EMT pathway via estrogen receptor (ERα) and GATA3 (233). Notch 4 signaling has been found to activate the EMT process in TNBC (234). However, Further evidence suggested that Jagged1-mediated activation of the Notch intracellular domain (NotchIC) through positive regulation of Slug suppresses E-Cadherin, resulting in EMT induction in breast malignancies (235). A report revealed a correlation between TGF-β and Notch activity. Elevated Notch signaling mediated by Smad3 upregulates the expression of Jagged1 and HEY1, thereby inducing upregulation of Slug expression and subsequently inhibiting E-cadherin (229).

### 3.4 TNF-α/NF-κB signaling pathway in EMT

TNF- α is a transmembrane protein having a molecular weight of 26 kDa. It is a crucial cytokine in inflammation, cellular homeostasis, and tumor progression (236). It promotes angiogenesis, invasion, and metastasis related to EMT reprogramming by activating MMP 9 and preventing E-cadherin. The upregulation of TWIST 1 is associated with the induction of TNF-α in EMT. The upregulation of TNF-α showed an association with enhanced metastatic potential and invasiveness of BCa cells (237). Recent findings suggest that TWIST 1 activity is essential in promoting mouse BCa cell metastasis (238). Recent studies have shown that prolonged exposure to TNF-α activates NF-κB and IKK-β, leading to EMT and the transcriptional repressor TWIST 1 and cancer stemness (239). The direct association between expression of TNF-α by peripheral blood T lymphocytes and EMT markers present in circulating tumor cells is reported (240). The activation of NF-κB is associated with Snail, Slug, TWIST, ZEB1/2, and NF-κB activation (236). The study has revealed the activation of NF-κB leads to Snail stabilization by degrading GSK-3β in the TNF-α/NF-κB activation pathway (236). Similarly, vimentin and MMPs of mesenchymal cell markers are also activated by NF-κB (241).

### 3.5 Hedgehog pathway in EMT

The hedgehog (Hh) pathway, associated with stem cell renewal, is another signaling system involved in the EMT of BCa. It also requires tissue homeostasis and embryonic development (242). Three glioma-associated oncogenes (GLI) transcription factors, GLI1, GLI2, and GLI3, have a role in either inhibiting or activating the transcription of these components in the Hh pathway (243). The role of the Hh pathway in EMT-derived BCa has already been established. The high expression level of GLI1 in BCa cells attaining EMT has already been reported (244). The role of the Hh pathway in cancer cell stemness and the interplay between NF-κB and GLI1 is also studied. Like the Wnt pathways, it is regulated through canonical or non-canonical signaling. According to a study, it has been revealed that non-canonical activation of GLI1 by hypoxia or other inflammatory cytokines can lead to the induction of EMT, BCa invasiveness, and drug resistance (245). The expression of GLI1 and its role in EMT in BCa via the Hh pathway has been confirmed through *in vivo* studies (246).

### 3.6 PI3K-AKT signaling pathway in EMT

Several RTKs have been identified for their role in the EMT of BCa cells (207). There are various factors involved in the activation of RTKs, including hepatocyte growth factor (HGF), epidermal growth factor (EGF), and fibroblast growth factor (FGF). HGF has a role in epithelial differentiation upon downregulating E-cadherin, which is responsible for tumor metastasis. The HGF pathway is also linked to the Snail transcription factor, which induces EMT (207). MAPK and PI3K are two signaling pathways along with TGF-β control invasion and EMT in BCa (197). Ras-activated MAPK stimulates TWIST 1 serine 68 phosphorylation and stabilization of PI3K signaling, which causes EMT and invasion of BCa cells (247). Recent studies indicated a possible connection between RTK, Wnt, and EGFR signaling (248). Though studies revealed the importance of RTK signaling and its role in EMT, various signaling pathways are also involved in EMT. The activation of the RTK pathway alone is not enough to induce EMT; multiple pathways are also involved.

## 4 Prospects and challenges

The inhibition of EMT inducers is necessary for metastasis and migration suppression (32). The strategies to induce differentiation and target EMT alone may result in adverse effects via the proliferation of metastatic cells. Therefore, targeted therapies combining various EMT marker molecules involved in different cell cycles are the novel approach. Although biomarker genes, miRNA, lncRNA, and multiple proteins of the TNBC secretome have made significant advances as therapeutic targets for particular tumors and as a potential molecular indicator for early clinical detection, there is still much to understand about their proneness of specific drug resistance and new strategies to target the TNBC (249). However, recent studies have shown that these biomarker genes are essential for tumorigenesis and progression of several tumors, including BCa, particularly TNBC, which exhibits challenges to available therapy due to its aggressive behavior (214). A substantial amount of data shows that differential expression of these genes has been significantly associated with BCa subtypes (250). It is well known that the need for effective targeted drugs to improve the survival rate of patients with distant metastasis is discouraging (251). The identification and function of metastasis-related molecular markers such as miRNA and lncRNA and proteins will lead to significant progress.

## 5 Conclusions

**Figure 5:**
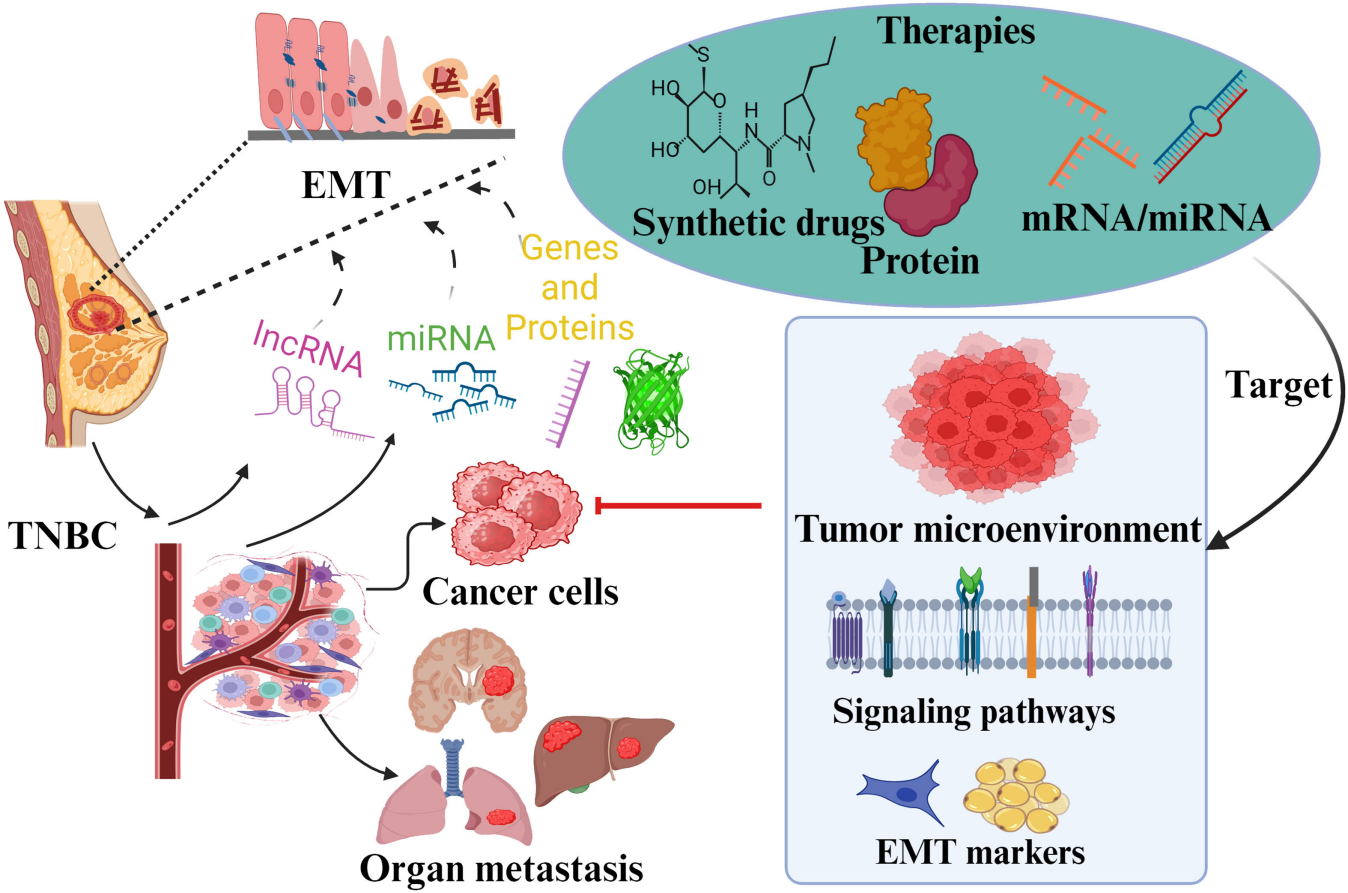
Overview summary shows EMT’s role and associated factors in TNBC pathogenicity. Various transcription factors and markers involved in TNBC metastasis due to EMT are presented. These factors have been discussed as potential approaches for targeted therapy. The role of different signaling pathways in EMT regulation is also reviewed to give insights into specific drug targets against TNBC.

Tumor cell invasion, metastasis, and EMT are responsible for the development of advanced-stage BCa, which needs an effective approach to reduce the tumor burden and improve the patient’s survival. Multiple signaling pathways and factors work together to make these complicated systems operate in BCa patients. This review describes the factors involved and the regulation of signaling pathways **Figure 5**. Evidence from studies shows that EMT is linked not only to tumor cell invasion and metastasis but also can give tumor stemness properties and induce drug resistance in the cells (36). Consequently, cancer cells with an EMT phenotype show more aggressive behaviors, such as drug resistance, stress, apoptosis, suppression of senescence, immunological evasion, and the acquisition of stem cell-like characteristics, in addition to their mesenchymal characteristics. The association of tumor EMT with autophagy and the surrounding microenvironment has been revealed from the studies. Inflammation, immune cells, tumor-associated fibroblasts, extracellular matrix, signaling chemicals, excessive acidity, and low oxygen are related to the tumor microenvironment. For example, the inflammatory microenvironment within the tumor can result in the EMT phenotype of the tumor cells. Hypoxia induction and inflammatory factors in the tumor microenvironment simultaneously trigger EMT, and the tumor microenvironment is also involved in the process of EMT. Therefore, EMT and the tumor microenvironment interact and affect each other to enhance tumor metastasis.

Additionally, since autophagy plays a role in tumor cell invasion and metastasis, a strong correlation has been shown between the emergence of EMT and autophagy. Tumor cells increase the production of autophagosomes, which contributes significantly to the development of EMT and increases tumor resistance against the immune system. Therefore, a strategic approach of combining different agents that can target EMT at multiple levels along with minimum side effects would aid in developing EMT as a successful novel target for tumors and cancer therapy.
